# HELP! Problems in executing a pragmatic, randomized, stepped wedge trial on the Hospital Elder Life Program to prevent delirium in older patients

**DOI:** 10.1186/s13063-017-1933-4

**Published:** 2017-05-17

**Authors:** Noor Heim, Henk F. van Stel, Roelof G. Ettema, Roos C. van der Mast, Sharon K. Inouye, Marieke J. Schuurmans

**Affiliations:** 1 Julius Center for Health Sciences and Primary Care, University Medical Center Utrecht, Utrecht, The Netherlands; 2 Research Center for Innovations in Health Care, Faculty of Health Care, Utrecht University of Applied Sciences, Utrecht, The Netherlands; 3 Department of Psychiatry, Leiden University Medical Center, Leiden, The Netherlands; 4 Department of Psychiatry, CAPRI-University of Antwerp, Antwerp, Belgium; 5 Department of Medicine, Beth Israel Deaconess Medical Center, Harvard Medical School, Boston, MA USA; 6 Aging Brain Center, Institute for Aging Research, Hebrew SeniorLife, Boston, MA USA

**Keywords:** Stepped wedge trial, Pragmatic trial, Delirium

## Abstract

**Background:**

A pragmatic, stepped wedge trial design can be an appealing design to evaluate complex interventions in real-life settings. However, there are certain pitfalls that need to be considered. This paper reports on the experiences and lessons learned from the conduct of a cluster randomized, stepped wedge trial evaluating the effect of the Hospital Elder Life Program (HELP) in a Dutch hospital setting to prevent older patients from developing delirium.

**Methods:**

We evaluated our trial which was conducted in eight departments in two hospitals in hospitalized patients aged 70 years or older who were at risk for delirium by reflecting on the assumptions that we had and on what we intended to accomplish when we started, as compared to what we actually realized in the different phases of our study. Lessons learned on the design, the timeline, the enrollment of eligible patients and the use of routinely collected data are provided accompanied by recommendations to address challenges.

**Results:**

The start of the trial was delayed which caused subsequent time schedule problems. The requirement for individual informed consent for a quality improvement project made the inclusion more prone to selection bias. Most units experienced major difficulties in including patients, leading to excluding two of the eight units from participation. This resulted in failing to include a similar number of patients in the control condition versus the intervention condition. Data on outcomes routinely collected in the electronic patient records were not accessible during the study, and appeared to be often missing during analyses.

**Conclusions:**

The stepped wedge, cluster randomized trial poses specific risks in the design and execution of research in real-life settings of which researchers should be aware to prevent negative consequences impacting the validity of their results. Valid conclusions on the effectiveness of the HELP in the Dutch hospital setting are hampered by the limited quantity and quality of routine clinical data in our pragmatic trial. Executing a stepped wedge design in a daily practice setting using routinely collected data requires specific attention to ethical review, flexibility, a spacious time schedule, the availability of substantial capacity in the research team and early checks on the data availability and quality.

**Trial registration:**

Netherlands Trial Register, identifier: NTR3842. Registered on 24 January 2013.

## Background

The evaluation of the effects of complex interventions in health care can be challenging. Complexity of an intervention is determined by the number of (independently and interdependently acting) components, behaviors and actors targeted, and the degree of flexibility and tailoring of the intervention [1, 2]. Complex interventions are considered to be difficult to standardize and to be sensitive to the features of the local context. Therefore, conventional experimental methods are not always suitable to evaluate complex interventions. In the current article, we report on the lessons learned performing a pragmatic, randomized, stepped wedge trial concerning a complex intervention for quality improvement in hospital care for older people in The Netherlands.

Our decision to perform a stepped wedge randomized trial was based on assumptions favoring the design over other options. In general, this design is in favor when there is already evidence in support of the intervention (for example, known to be effective at the individual level but uncertainty at the policy level), or when there is resistance to a parallel design in which only half of the clusters receive the intervention [3–5].

The Hospital Elder Life Program (HELP) [6] involves the implementation of practical tailored interventions to prevent delirium, targeting reorientation, early mobilization, therapeutic activities, hydration, nutrition, sleep strategies and hearing and visual adaptations, by trained volunteers [7]. As such, the HELP is a complex intervention and has been shown to be effective in the prevention of delirium during hospital stay in several countries [8]. Because of differences in health care systems and patient populations, previous results on the effectiveness of the HELP could not automatically be extrapolated to the Dutch situation. Therefore, a trial was designed to study the effectiveness of the HELP in the Dutch hospital setting.

At the time the study was designed, we made assumptions on the advantages of the stepped wedge design. First, the hospitals involved in our study were already planning to start using the HELP. By using a stepped wedge design, all participating units would receive the intervention and would be assisted in the implementation process by the study team. Second, the stepwise implementation of the intervention enabled the phased recruitment and training of volunteers, thus enhancing feasibility in practice. Third, as is the case for most quality improvement efforts, especially when routinely collected data are used, we expected that obtaining written informed consent pretreatment would not be required [9]. Fourth, the external validity and generalizability were expected to be optimal given the anticipated absence of the need for individual recruitment of study participants [10]. Fifth, a known drawback of the chosen design was the inability to blind study staff, potentially causing selective inclusion and reduced reliability of the primary outcome. This drawback was minimized by extensive training of the nurse practitioners (NPs) responsible for measuring the primary outcome [5].

In the current paper, we report on the experiences and the problems faced during the conduct of the pragmatic, stepped wedge trial on the HELP and lessons learned for future studies. We use the results of our trial on the effects of the HELP to illustrate the processes.

## Methods

We started evaluating our trial focusing on the assumptions about the design that we had at the start of our study. We reflected on our intentions for the execution of the trial and how these compared to what we in fact realized and from this comparison we drew the lessons learned. The contemplation and description of the experiences and problems faced during the execution of the trial and the lessons we learned were derived from extensive surveys and structured discussions among the co-investigators,The﻿se investigators ﻿were experienced researchers regarding complex interventions, with various backgrounds including methodological as well as content expertise. Below, the design of the HELP trial is described to facilitate understanding of the experiences, problems and lessons learned.

### Design of the pragmatic, stepped wedge trial

**Fig. 1:**
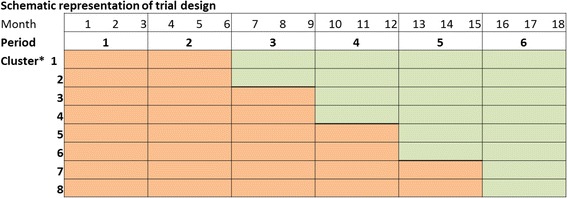
Schematic representation of the planned stepped wedge, cluster randomized trial study design. *Orange boxes*, cluster not exposed to intervention. *Green boxes*, cluster exposed to intervention. * Participating units were to be randomized to clusters 1–8 and would all start with a control period. Every 3 months, two units would cross over to use the intervention

The methods and design of the randomized, stepped wedge trial assessing the effects of the HELP in the Dutch setting have been comprehensively described in a previously published design paper [11]. The intervention study used a stepped wedge design according to the scheme displayed in Fig. 1. Eight units of two hospitals located in the center of The Netherlands were enrolled in the study from the start (cardiology, geriatrics, internal medicine and orthopedics and surgery at both hospitals). In one of the hospitals the internal unit and the surgery unit failed to include patients. To make up for the loss of these units, two units (cardiology and geriatrics), in a university hospital, were added to the trial in a later stage of the study. In an order randomly assigned using Excel, the units participating in our study consecutively started using the intervention during the study period.

### Study population

Eligibility to participate was assessed in all patients aged 70 years and over who were admitted to the participating units of the hospitals. Further inclusion criteria were the absence of delirium at the time of hospital admission and being considered at increased risk for delirium according to the three questions of the mandatory Dutch Hospital Safety Program (see below). Exclusion criteria were an expected hospital stay of less than 24 h or having a life-threatening condition, suffering from serious cognitive limitations, being legally incapable of participating, unable to communicate verbally, or receiving palliative care at the time of admission.

The a priori sample size needed was 940 patients equally distributed over the control group “pre-HELP” and the intervention group “the HELP condition” (see design paper [11] for the full sample size calculations) [8]. The sample size calculation was based on reduction of 10% in the primary endpoint incidence of delirium. To demonstrate this difference, using a two-sided test with an alpha of 0.05 and a power of 0.90, two groups of 470 patients were required, indicating a study population of 940 patients. Taking into account 15% not willing to participate, the total number of eligible patients required was estimated at 1081 patients. The study achieved inclusion of 518 patients, and was thus underpowered to assess the study outcomes. When designing this study, not much was known yet about sample size calculations for stepped wedge designs. So, the power calculation was done for a simple randomized controlled trial, not taking clustering into account, making the study even more underpowered. When analyzing the data, we conducted a state-of-the-art analysis by taking the clustering into account in multilevel analysis. This way, the influence of a specific unit on the outcome of an individual patient is separately modeled, resulting in a less biased effect estimation of the intervention in the individual patient.

### Assessment of risk for delirium

As part of routine care, the risk for delirium was assessed by a nurse within 24 h after hospital admission using the three questions of the Hospital Safety Program [12]. This program was launched in The Netherlands in 2009 as part of mandatory hospital care for patients aged 70 years and older. Patients are considered to be at risk for delirium if one of the following questions is answered positively: “Do you have memory problems?”; “During the past 24 h, did you need assistance with your daily self-care?”; and “Have you ever been confused during earlier hospital admissions or illnesses?” If a patient was considered to be at risk for delirium, the patient was approached for study participation by a NP specializing in the care for older patients. The NPs were explicitly instructed to apply the same criteria for approaching and including patients in the pre-HELP and the HELP periods.

### Intervention

For an extensive description of the HELP intervention, we refer to the design article of our study [11] and to the articles in which the development of the original HELP is described [7, 13]. In close collaboration with the developer of the HELP, Dr. Sharon Inouye, all materials were adapted and translated into Dutch. In short, the components of the program were the provision of standardized protocols targeting risk factors for delirium, the introduction of elderly care NPs, bedside interventions conducted by trained volunteers, and regular reassessment of enrolled patients to keep personalized interventions matching the changing needs throughout the course of hospitalization. If, during the course of hospitalization, patients requested no volunteers, they did not receive further bedside interventions, but were not withdrawn from the study.

### Measurements

As much as possible, we relied on measurements done as part of routine clinical practice which were retrieved from electronic patient records at the end of the study. Telephone interviews were used to collect follow-up data on rehospitalization and admission to residential care.

#### Primary outcome: delirium

According to Dutch guidelines, nurses had to administer the Delirium Observation Screening Scale (DOSS) [14] as part of routine care three times daily for three consecutive days after a patient screened positive for an increased risk for delirium. When a positive DOSS occurred, incident delirium was confirmed by the NP or geriatrician using the Confusion Assessment Method (CAM) [15]. The CAM includes a four-item diagnostic instrument for delirium assessing the acute onset and fluctuating course of inattention (i.e., distractibility), and either disorganized thinking (i.e., illogical or unclear ideas) or an alteration in consciousness.

#### Secondary outcomes

The secondary outcomes were hospital length of stay, change in health-related quality of life [16], hospital readmission, and admission to an institution assessed monthly for 3 months after discharge in a random subset of study patients. Hospital length of stay was determined as a secondary outcome from the electronic patient records. Additionally, patients were contacted monthly by telephone for 3 months after discharge to assess readmission to the hospital or admission to an institution (nursing home or rehabilitation facility). If patients were not able to participate, a close family member was asked for information on admission to a hospital or institution.

#### Covariates assessed at baseline

From the electronic patient records, level of education and Activities of Daily Living (ADL) functioning were extracted. ADL function at baseline was assessed using the Katz Index on independence in ADL [17], a six-item instrument to assess independence. Trained NPs additionally assessed cognitive function, using the Six-item Cognitive Impairment Test (6CIT), and self-rated health using a Visual Analog Scale asking patients to rate their own health on a scale of 0 to 100 [18, 19].

### Statistical analysis

Using multilevel regression models, the incidence of delirium (logistic), the change in health-related quality of life during hospital stay (linear), the length of stay (Poisson), (re-)hospitalization (logistic) and admittance to an institution following discharge (logistic) were compared between the pre-HELP (control) and HELP (intervention) patient groups. All analyses were adjusted for the clustering of patients within units, period effect and for baseline characteristics (age, sex and ADL function at baseline). All statistical analyses were performed using the lme4 package [20] for R, version 3.1.3 [21].

## Results

In the results section, we will show the results of the HELP trial as an illustration of the problems experienced and the lessons learned. The results, experiences and lessons learned are organized in three areas: (1) inclusion of patients, (2) use of data collected during routine care and (3) performance of the stepped wedge design. An overview of the experiences, the problems faced and the lessons learned can be found in Table 1.

**Table 1:** Overview of the lessons learned from the execution of our pragmatic, cluster randomized, stepped wedge trial

Aspects of study design	Assumptions/considerations	Intended	Realized	Lessons learned
Cluster randomized (stepped wedge) controlled trial design	The design promotes hospitals to participate because all participating hospitals will receive the intervention (which was shown effective in previous studies)	Participation of eight units in three (locations of) different hospitals, all starting with a (control) pre-HELP period	Two of the eight units were excluded from participation because of problems with the inclusion of patients during the pre-Hospital Elder Life Program (pre-HELP) period	Emphasize that the study starts before implementation of the intervention and the equal importance of accurate data collection both before and after the intervention is implemented
			The start of the trial was delayed. It took more time than expected to make all necessary arrangements, because not all hospitals were familiar with the regulations for performing a scientific study in the clinic	
				Allow for enough time to explain and familiarize participating hospitals with rules and regulations of scientific studies and make timely arrangements accordingly
			Because of the delay, the time frame available to complete our research project was no longer sufficient to stick to the original scheme. Therefore, we decided to start the intervention in period 1 in the first two units to make up for the delay	A stepped wedge, randomized trial takes more time than a regular cluster randomized trial. Be sure the time frame available is comfortably sufficient, even if difficulties occur
	Individual recruitment and informed consent of patients is not necessary when using routinely collected data for a quality improvement trial	Analyses of the effects according to the intention-to-treat principle in all eligible patients admitted to the participating units using data from the electronic patient record systems to reduce the risk of bias	The Medical Ethical Review Board required informed consent of every patient included and thereby made individual recruitment inevitable	When using a novel and/or rarely used study design, consider the need to discuss the assumptions underlying the design with the members of the Medical Ethical Review Board before submitting the research proposal
				Be sure to have a timely decision of the Medical Ethical Review Board to be able to adapt procedures to the need for individual recruitment
	The study design enables a gradual recruitment of volunteers, implementation of the intervention and inclusion of sufficient numbers of patients in each period in each unit	Recruit and educate volunteers in four cycles, to enable two new units to start the intervention every 3 months	The recruitment and education of volunteers went accordingly to plan	
		In the months before starting the intervention, nurses on the participating units include patients in the pre-HELP condition applying the same criteria to be used during the HELP period. Each unit had to include both pre-HELP and HELP patients to be compared and to avoid empty cells in the stepped wedge matrix	Several units had difficulties including patients in the pre-HELP condition. Two units were excluded from the study after a couple of months in which no patients were included. The ultimate result is the presence of empty wedges in the stepped wedge matrix	Strongly emphasize the importance of including patient during the preintervention period in every participating unit
Inclusion of eligible patients	No need for informed consent	We intended to study the effects of a safe, noninvasive quality improvement intervention	The Medical Ethical Review Board decided informed consent was required. Inclusion of patients in our study severely lagged behind the expected numbers. The requirement of informed consent is likely to have diminished the number of patients willing to participate	Be sure to have a timely decision of the Medical Ethical Review Board to be able to adapt procedures
			The Ethical Committee permitted the use of a delayed informed consent procedure to improve the participation rate in a later stage.	
	No need for individual recruitment of patients	By applying the intervention to all patients at risk and using routinely collected data to monitor the effects, results of the intention-to-treat analyses are maximally generalizable	The need for informed consent and, therefore, individual recruitment in our nonblinded study, made the inclusion more prone to selection bias and the results less generalizable.	Reconsider the use of a stepped wedge, cluster randomized trial when there is a need for individual consent
			The need for individual inclusion of the eligible patients made the analyses of the effects according to the intention-to-treat principle on cluster level to reduce the risk of bias impossible	
	Applying the same inclusion criteria in the control condition and in the intervention condition will create a balanced study population without empty cells in the stepped wedge matrix	Similar (numbers of) patients will be included in the wedges in the control period as compared to the intervention period	Comparing the baseline characteristics of patients included in the control versus the intervention period showed that patients included in the HELP condition seem slightly healthier. This may imply selective inclusion favoring positive effects of the HELP	Especially when the recruitment is done by different persons over the units, education on using the same inclusion criteria when considering patients for inclusion in both the control and the intervention condition is crucial
		Each unit will include both pre-HELP and HELP patients to be compared and no empty cells will occur in the stepped wedge matrix	We failed to include a similar number of participating patients in the control condition versus the intervention condition. This caused the dropout of two units, empty cells in the stepped wedge matrix and an unequal distribution of patients in the control and the intervention groups. These phenomena would partly be concealed in a regular cluster randomized trial	Constant monitoring and motivational activities should be executed to enhance inclusion of patients, especially during the control period
Use of routinely collected data	Helps to avoid selection bias (especially in the absence of the need to individually recruit patients) and enhances external validity because of the optimal reflection of usual situation	Routinely collected data from electronic patient record systems would be consecutively made available over the time frames of the stepped wedge design.	Data from the electronic patient records were not accessible during the study, but only provided after the study period was finished	Check the availability, consistency and quality of routinely collected data well before the study and monitor data quality throughout the study
		According to the study protocol, nurse practitioners (NPs) were to confirm the diagnosis of delirium using the Confusion Assessment Method (CAM) if the results of the routinely collected data on the Delirium Observation Screening Scale (DOSS) were positive	When receiving the data, a lot of data on the outcome appeared to be missing. As a consequence, an important share of the patients included in the study could not be included in the analyses, causing more empty cells in the stepped wedge matrix	
	Data crucial for the assessment of the effectiveness of the HELP were considered to be present in all patients eligible for the study (who all were at increased risk for delirium)			Assure timely accessibility of the data to be able to do early checks on the quality and completeness of the data during the study period

**Fig. 2:**
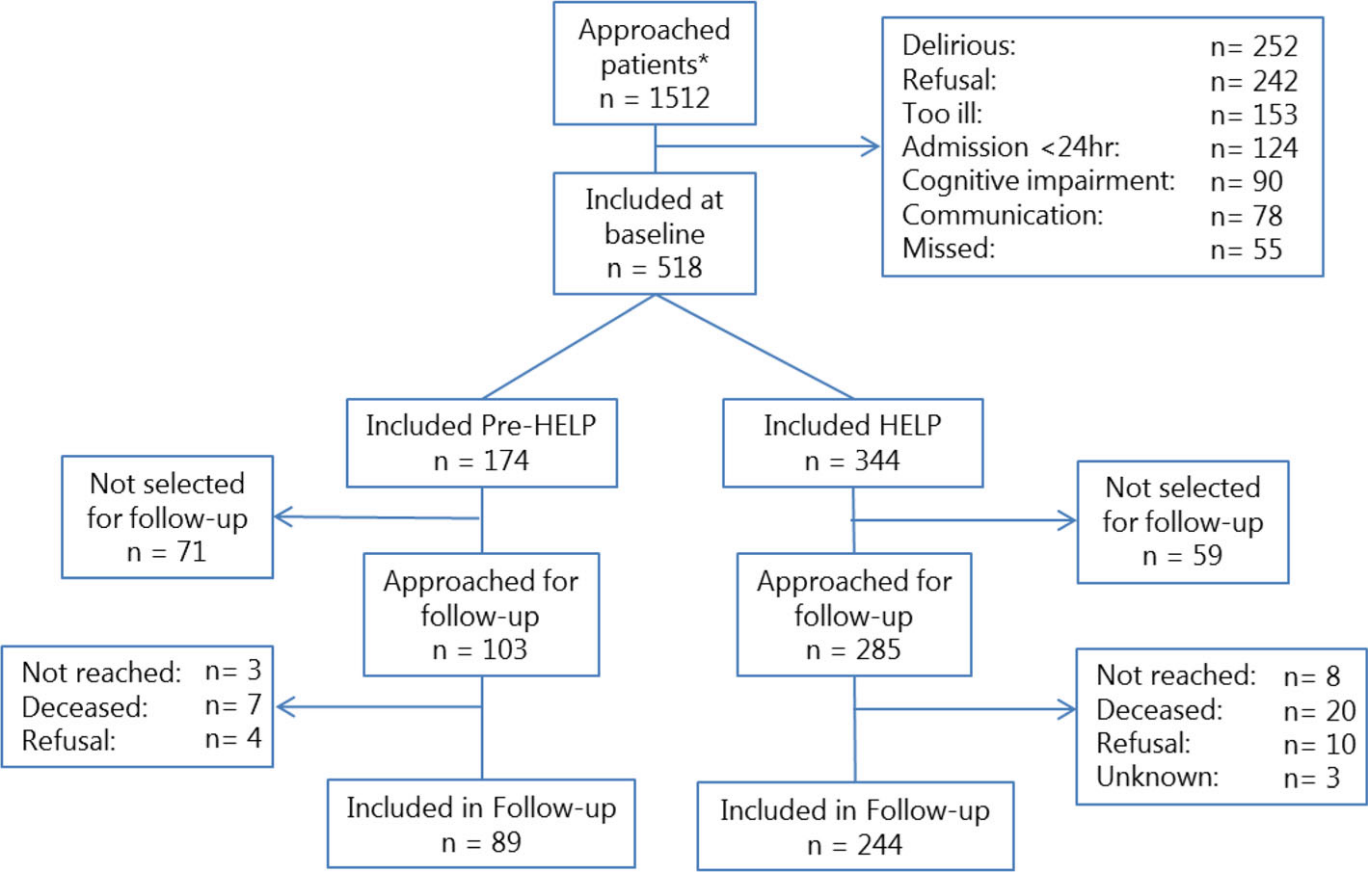
Flow chart of the patients approached for participation and the study population included pre-Hospital Elder Life Program (pre-HELP) and during the HELP intervention and follow-up. * It is unknown whether patients were approached in the pre-HELP or in the HELP period

**Fig. 3:**
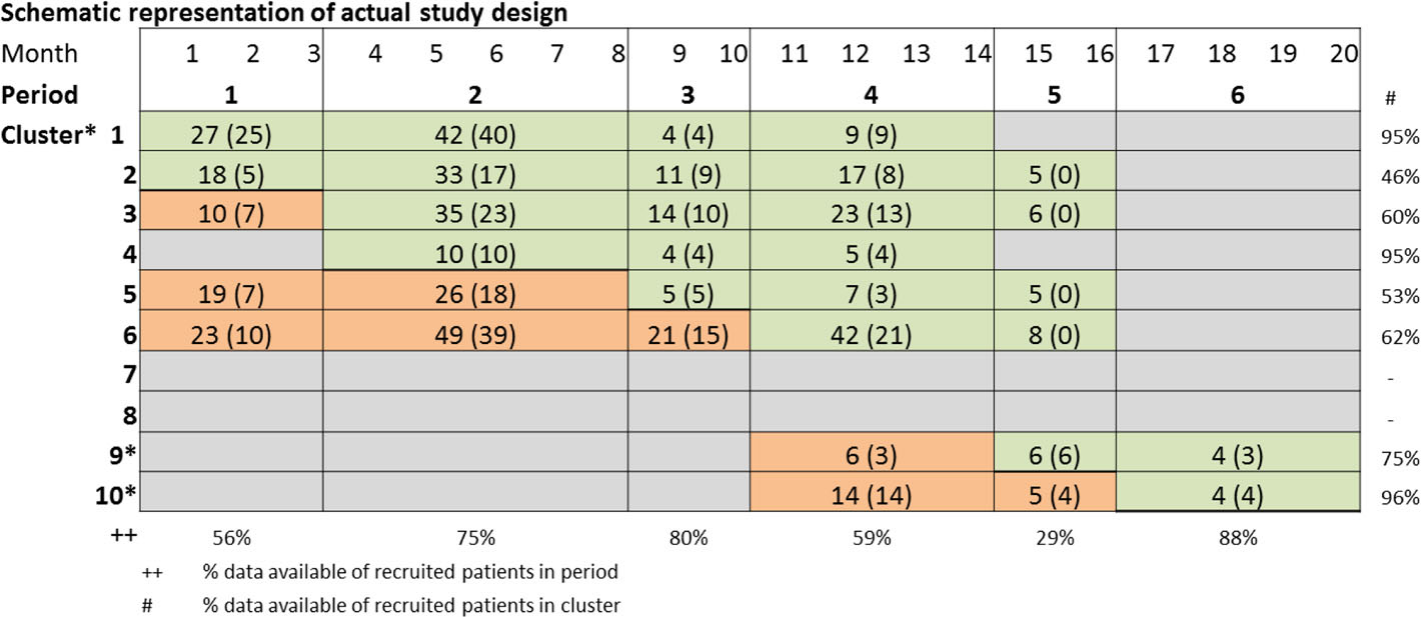
Schematic representation of the inclusion of patients per period in each cluster in the actual study design of the stepped wedge, cluster randomized trial. *Orange boxes*, cluster not exposed to intervention. *Green boxes*, cluster exposed to intervention. *Beige boxes*, cluster in which no patients were included in the specified period. * Participating units 1–10 were randomized to clusters subsequently starting to use the intervention

1. *Results of the inclusion of patients in the HELP stepped wedge trial*
  The results of the inclusion in the baseline and the follow-up measurements per protocol are shown in the flow chart in Fig. 2. The scheme in Fig. 3 displays the enrollment of patients in each period and in each of the clusters of the trial. The baseline characteristics of the study population are displayed in Table 3 of the Appendix.

### Experiences with the inclusion of eligible patients

The Medical Ethical Review Board unexpectedly required written pretreatment informed consent from every individual patient, whereas we intended to provide a safe quality improvement intervention, requiring only post-treatment consent for using existing clinical data and completion of a short questionnaire. The study was already designed, planned and prepared when the decision concerning the requirement for pretreatment informed consent was made. More time between the Medical Ethical Review Board’s decision and the start of the study would have given us the opportunity to consider adapting our procedures or time schedule to still be able to enroll sufficient numbers of patients. The required written informed consent most likely decreased the number of patients enrolled into our study and might have also introduced a selection bias. Especially in the patient population that HELP aims to support, requesting full informed consent from patients who often do not have a full overview of their situation, has a negative impact on recruitment. This consent problem is not unique to stepped wedge designs, but the impact on low and unequal accrual in the wedges is unique.

From the start, the enrollment of patients lagged behind the expected numbers. After a study period of 6 months in which no patients had been included on two of the eight units, these units were eliminated from the study. The training of the NPs responsible for the inclusion of patients might not have emphasized strongly enough the importance of accurate data collection on all eligible patients in the pre-HELP period. Especially during the preintervention phase, constant monitoring and motivating activities were necessary. Allowing time for a run-in phase, to assure that all staff on participating units are familiar with the inclusion criteria, procedures and measurements, could help to improve enrollment and minimize missing data in the preintervention (control) phase of the trial. Two units of a third hospital were added to the study in a later stage, to make up for the loss of eligible participants on the two units that were dropped from the study. Also, more patients than expected were unable or unwilling to participate. The proportion of patients who were delirious at the time of inclusion was higher than accounted for in the power calculations. The estimation of the number of prevalent cases of delirium at the time of inclusion was based on the registration of delirium at the time of admission in the months prior to the start of the study. This registration took place during the anamnesis soon after the patients arrived in hospital, while the inclusion of patients took up to 72 h. The early incidence of delirium was not adequately considered in the estimation of the number of patients to be excluded because of a prevalent delirium

2. *Results on the use of routinely collected data in the HELP stepped wedge trial*
  When the data from included patients were retrieved from the medical records, it was found that many data on the outcome were missing. The DOSS should have been available in all included patients; however, the data were missing in 194 (37%) of the patients. When a positive DOSS occurred, incident delirium was to be confirmed by the NP or geriatrician using the CAM. However, CAM scores were missing in 48 of 64 patients (75%) with a positive DOSS score. The CAM was also assessed in 15 patients from whom no DOSS score was available. We decided to use a combination of both DOSS and CAM scores. Patients with a positive CAM and/or a mean DOSS score of 3 or higher, in at least two assessments within 1 day in the first 5 days of hospital admission, were considered to have an incident delirium.
  Even using this adapted assessment of the outcome measure, 34% of the included patients (*n* = 178) had missing data and could not be included in the final analyses of the incidence of delirium in the HELP trial. The patients with complete data were unequally distributed over the pre-HELP and the HELP groups in the trial. In total, data on incident delirium were available for 117 patients included in the pre-HELP control period; and 223 patients included during the HELP intervention period. Between the brackets in each of the cells of the matrix in Fig. 3, the number of patients with complete data on incident delirium in each period and in each of the clusters are displayed. As can be seen, the availability of complete data varied widely over the periods (29–88%) and the clusters (51–100%)

### Experiences with the use of routinely collected data

Prior to the study, extracting routinely collected data on the outcomes from electronic patient records presented a cost-efficient and pragmatic approach. When the study started, all hospitals had (recently) introduced electronic patient record systems and all were optimistic about the feasibility of the extraction of data from the systems. However, these data were far less accessible than expected and retrieval of data was highly delayed. We received data in a late stage of the study, when it was already too late to adapt procedures to ensure the availability of data on the outcome. Data that should be available according to clinical protocols, were either not collected or not recorded in the medical record.

In a classical trial design, missing values can be imputed based on the total number of patients in the intervention group and in the control group. For our stepped wedge design, however, because of there being too few patients in each cell of the stepped wedge matrix, in combination with the presence of empty cells, imputation of missing values could not be done. We learned that checking the availability, consistency and quality of routinely collected data before the study, and performing ongoing quality checks on the data throughout the study are essential.

3. *Results on the effectiveness of the intervention ﻿and on the perfomance﻿ the HELP stepped wedge trial*

**Fig. 4:**
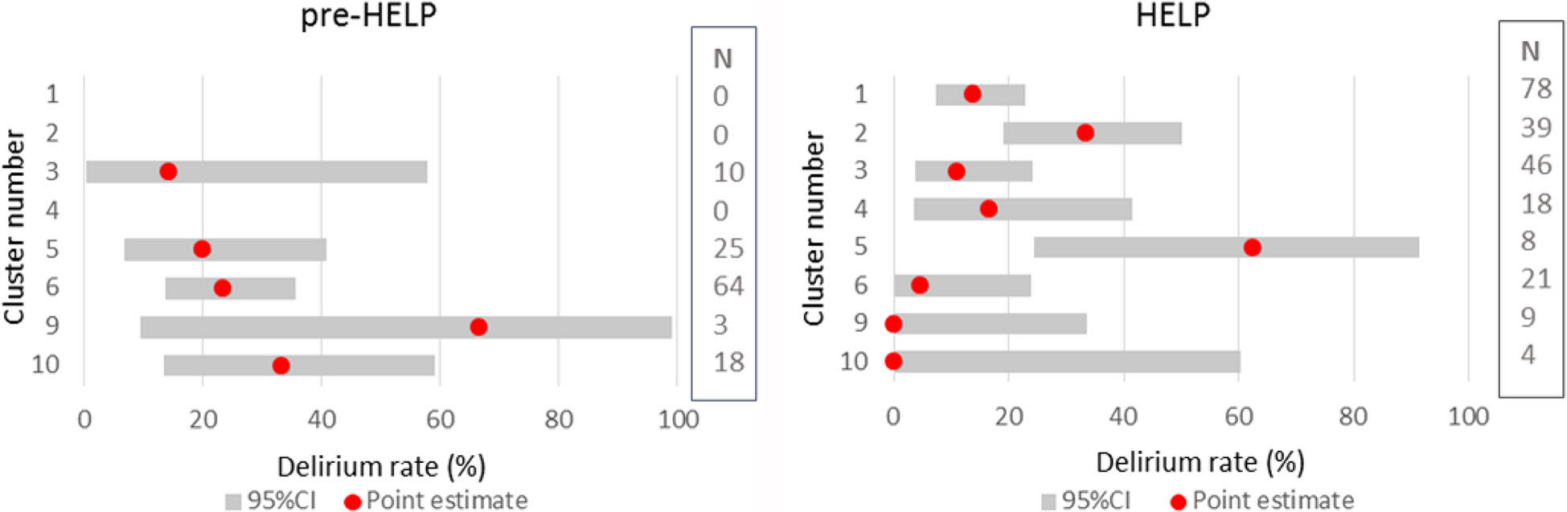
Estimates of the incidence of delirium in each cluster during the pre-Hospital Elder Life Program (*pre-HELP*) (*left panel*) and the HELP (*right panel*) periods

In Table 2, the incidence of delirium is shown for the patients included in the pre-HELP group and for those included in the HELP group, as well as the results on the secondary outcomes. The incidence of delirium in the pre-HELP period was 23.9%, whereas the incidence during the intervention period was 15.2%. The multilevel analyses of these data showed a nonsignificant adjusted odds ratio for delirium in the HELP period of 0.51 (95% CI 0.22–1.19) as compared to the pre-HELP period. The estimate of the risk reduction was bigger than accounted for when designing the study, but the inadequate sample size caused a lack of power. Furthermore, stratifying the incidence of delirium by cluster revealed large intracluster variation in both the pre-HELP and the HELP periods which might add to the lack of statistical significance of the odds ratio. Figure 4 visually demonstrates the variation of the incidence of delirium per cluster in the pre- HELP and the HELP periods. None of the secondary outcomes were significantly different between the groups.

**Table 2:** Results of the multilevel regression analyses on the outcomes for the pre-Hospital Elder Life Program (pre-HELP) and HELP groups

	Pre-HELP		HELP		GLM^a^	
	*n*		*n*		estimate	Confidence interval
During hospital stay						
Incident delirium (%)	117	23.9	223	15.2	OR = 0.51	0.22–1.19
LoS, median (IQR)	151	9.0 (7–12)	332	9.0 (6–13)	RR = 1.06	0.97–1.16
Change EQ-5D, mean (SD)	100	0.13 (0.32)	235	0.13 (0.29)	*β* = −0.01	−0.09–0,06
At 3-month follow-up						
(Re-)admittance to hospital (%)	80	21.3	237	24.9	OR = 1.24	0.65–2.37
Admittance to institution (%)	78	43.6	232	37.9	OR = 1.06	0.59–1.90

*LoS* length of stay, *EQ-5D* EuroQol 5 dimensions quality of life questionnaire, *SD* standard deviation, *IQR* interquartile range, *GLM* General Linear Models, *OR* odds ratio, *RR* risk ratio

^a^Incident delirium, (re-)admittance to hospital and admittance to institution were analyzed using logistic, LoS using Poisson and change in EQ-5D using linear regression multilevel models. All GLM analyses were adjusted for age, sex and baseline Activities of Daily Living (ADL) and for clustering and time effect

### Experience with the performance of the stepped wedge design

It took more time than expected to obtain the necessary (human subjects/ethical clearances) arrangements in all units, which caused a delay of the start of the trial. Because of the strict time frame for our research project, we had to make concessions to be able to carry out the relatively time-inefficient stepped wedge trial within the permitted time frame. It was, therefore, decided to immediately start with the intervention on two units (clusters 1 and 2). We hoped that the successful inclusion of patients in the control condition in the other clusters would make up for the absence of control patients in the clusters where we started the intervention. Additionally, we also failed to include patients in the pre-HELP period in one of the other clusters. Therefore, no comparison with usual care could be made in three clusters. Furthermore, the high variability in both baseline and change in delirium incidence negatively influences our belief in the robustness and generalizability of the change estimate. The nonblinded administration of the required DOSS by nurses could be a contributing factor in this variability, due to observer bias. The teams to perform the intervention in new wedges were recruited, educated and ready to start on a timely basis. However, with disappointing numbers of patients included, moving on to the next phase of the trial caused imbalance in the size of the pre-HELP versus the HELP groups. In our experience, the stepped wedge design is relatively rigid and leaves little space to improvise and adapt enrollment procedures. We learned that planning far ahead and the availability of substantial capacity in the research/implementation team are essential.

## Discussion

We faced several challenges in the execution of our pragmatic, randomized controlled, stepped wedge trial. The problems faced caused inadequate sample size and the incomplete assessment of the outcome and potential selection bias due to the individual recruitment of patients. Conclusions on the effectiveness of HELP in the Dutch hospital setting could, therefore, not be drawn. We did, however, learn important lessons during the conduct of our study that can help others to enhance their study planning and design. Although the stepped wedge trial is used since the early years of this century, reporting experiences with this design is scarce. A recent review of stepped wedge trials found a total of 123 studies, of which 39 were completed trial reports [3]. The quality of the reporting is these trials varied and the authors concluded that there is much room for improvement. None of the existing reviews on methodological aspects of stepped wedge trials discuss the problems encountered when executing such a study. We identified a paucity in papers describing the practical challenges of executing stepped wedge trials and their impact on conduct and analysis.

### Inclusion of eligible patients

Including sufficient patients in each phase has been proven challenging in other studies. A stepped wedge trial typically needs a longer duration than other cluster randomized design [22]. The strict scheme that researchers have to fulfill has previously been mentioned as a major drawback of the stepped wedge design [23, 24]. Faced with fewer than the required numbers of patients included in each step can lead to cumulative delays. When planning a trial, potential delays should be anticipated and taken into account before deciding to perform a stepped wedge trial. It is important to make sure that there can be time allowed for unforeseen delays and to alter study procedures as often happens in clinical trials.

### Use of routinely collected data

By relying on the medical record for collection of data, we wanted to mimic the real-life circumstances in the hospital units as closely as possible for our pragmatic trial. During the study, data were not yet retrieved from the medical records and we were not aware of the many missing data. Apart from the fact that a large quantity of data on the outcome were missing, uncertainty about the quality of these data also has important consequences on the interpretability. We cannot rule out the possibility that the available data were collected selectively. For example, the measurements might have been assessed by indication, such that nurses started assessing DOSS scores if they had the impression that a patient was starting to become confused or disoriented. This phenomenon has recently been described by IJkema and colleagues [25]. More generally, it has been shown that data from sicker patients tend to be more complete than those of less sick patients [26]. In the intervention group, relatively less data on the outcome were available. Characteristics of patients with complete data are similar between patients in the pre-HELP and the HELP group as compared to the whole study population, suggesting that the data are no more or less selective in either group. When relying on data collected in routine care, the quality and completeness of the data demanded should be established before the study starts and inspections of data collected during the trial should be incorporated at multiple timepoints [27]. Imputing missing values is less feasible in the stepped wedge design compared to the classical trial design because of the limited number of patients within the time slots of a stepped wedge trial. We could not impute missing values in our study due to both the insufficient numbers of patients per cell and the presence of empty cells caused by the problems with inclusion. Furthermore, by the time that a study is well on its way, it is difficult to adapt procedures to compensate for or minimize missing data.

### Performance of a stepped wedge trial

With our stepped wedge trial, we intended to pragmatically study the effectiveness of the HELP program in the Dutch context, which had not been previously investigated. Hospitals were already planning to roll out the program, which was expected to have beneficial effects (and unlikely to do any harm). These factors were recently considered sound justifications for conducting a stepped wedge trial [5, 22, 23]. The design has especially been recommended to evaluate service delivery interventions where outcomes are based on routinely collected data for which no individual recruitment is required [28]. We intended to evaluate service delivery according to the HELP using routinely collected data. However, we needed to individually recruit patients because pretreatment informed consent was demanded by the Medical Ethical Review Board. A lack of familiarity with our intervention procedures or study design may have factored into this decision. Providing more information on the assumptions, aims and characteristics of the design and intervention, along with their pros and cons, might have resulted in a different decision. Previous trial literature concerning quality improvement (research) projects in health care advocated that the ethical and methodological aspects differ significantly from other types of clinical research and the ethical appraisal of the design might warrant special expertise and a shift in priorities by Medical Ethical Review Boards [10, 29–31].

The disappointing inclusion rates and missing data on the outcome also caused the stepped wedge matrix to be unequally distributed which likely impacted the robustness of the results in our statistical analyses [24, 32]. At the time that our study was designed not much was known on the calculation of the sample size needed to reach sufficient statistical power in a stepped wedge trial. Knowledge on this has grown over the last couple of years, but still no consensus seems to have been reached on the topic [32–34].

The hospitals involved in our study were all eager to start working with the HELP and, being unaware of the missing data, we decided to proceed according to our schedule. Previous studies have warned of increasing risk of units dropping out of the study, especially units randomized to a late implementation wedge, when prolonging the study period [24, 35]. Furthermore, uncertainty remains on the consequences of unequal duration of the periods in the matrix for the interpretation of the results of statistical analyses [24].

## Conclusions

The stepped wedge, cluster randomized trial offers opportunities to test and implement additions to usual care in a real-life setting, thereby maximizing feasibility and generalizability of the results. Researchers should be aware of the pitfalls of the design and the execution of research in real-life settings to prevent negative consequences for the validity of their results. In our pragmatic, stepped wedge, cluster randomized trial, we faced high variability in estimates between clusters, under-enrollment and limited quality of the data collected and, therefore, subsequent valid conclusions on the effectiveness of the intervention under study could not be drawn. We learned that when conducting a stepped wedge, cluster randomized trial to test the effect of a complex intervention, timely assessment of the protocol by the Medical Ethical Review Board, a spacious time schedule, the availability of substantial capacity in the research team and early checks on timely data availability and data quality are essential.

## Appendix

**Table 3:** Baseline characteristics of the study population stratified in a pre-Hospital Elder Life Program (pre-HELP) and HELP group

	All participating patients (*N * = 518)				Patients included in the analyses of incident delirium (*n* = 340)			
	Pre-HELP (*n* = 174)		HELP (*n* = 344)		Pre-HELP		HELP	
	*n*		*n*		*n*		*n*	
Age, median (IQR)	174	85 (80–88)	344	82.5 (78–87)	117	86 (81.5–89)	223	83 (79–88)
Sex (% male)	174	35.1	344	43.0	117	34.2	223	42.6
Level of education	156		301		117		223	
Below secondary education (%)		37.5		43.2		36.3		42.2
Secondary/vocational education (%)		51.6		50.5		51.0		52.5
Higher education (%)		10.9		6.3		12.7		5.3
6CIT >10 (%)	173	23.1	341	23.5	117	27.4	223	25.3
Katz ADL	152		332		117		223	
Independent (%)		38.2		44.6		35.9		40.8
Moderately dependent (%)		35.5		40.4		39.3		41.7
Severely dependent (%)		26.3		15.1		24.8		17.5
EQ-5D, mean (SD)	171	.42 (.33)	340	.45 (.31)	117	.43 (.33)	223	.44 (.31)
Self-rated Health by VAS, mean (SD)	167	58 (19)	331	56 (19)	117	58 (17)	223	54 (19)

*IQR* interquartile range, *SD* standard deviation, *6CIT* Six-item Cognitive Impairment Test, *ADL* Activities of Daily Living, *EQ-5D* EuroQol 5 dimensions quality of life questionnaire, *VAS* Visual Analog Scale, ranging from 0–100

## Abbreviations

6CIT: Six-item Cognitive Impairment Test
ADL: Activities of Daily Living
CAM: Confusion Assessment Method
CI: Confidence interval
DOSS: Delirium Observation Screening Scale
HELP: Hospital Elder Life Program
NP: Nurse practitioner
OR: Odds ratio
